# The impact of gape on the performance of the skull in chisel-tooth digging and scratch digging mole-rats (Rodentia: Bathyergidae)

**DOI:** 10.1098/rsos.160568

**Published:** 2016-10-12

**Authors:** Andrew F. McIntosh, Philip G. Cox

**Affiliations:** 1 Centre for Anatomical and Human Sciences, Hull York Medical School, University of Hull, Hull, UK; 2 Centre for Anatomical and Human Sciences, Hull York Medical School, University of York, York, UK; 3 Department of Archaeology, University of York, York, UK

**Keywords:** cranial biomechanics, finite-element analysis, chisel-tooth digging, scratch digging, Bathyergidae

## Abstract

The African mole-rats (Bathyergidae) are a family of rodents highly adapted for life underground. Previous research has shown that chisel-tooth digging mole-rats (which use their incisors to dig burrows) are clearly distinguishable from scratch diggers (which only use the forelimbs to tunnel) on the basis of morphology of the skull, and that the differences are linked to the production of high bite forces and wide gapes. We hypothesized that the skull of a chisel-tooth digging mole-rat would perform better at wider gapes than that of a scratch digging mole-rat during incisor biting. To test this hypothesis, we created finite-element models of the cranium of the scratch digging *Bathyergus suillus* and the chisel-tooth digging *Fukomys mechowii*, and loaded them to simulate incisor bites at different gapes. Muscle loads were scaled such that the ratio of force to surface area was the same in both models. We measured three performance variables: overall stress across the cranium, mechanical efficiency of biting and degree of deformation across the skull. The *Fukomys* model had a more efficient incisor bite at all gapes, despite having greater average stress across the skull. In addition, the *Fukomys* model deformed less at wider gapes, whereas the *Bathyergus* model deformed less at narrower gapes. These properties of the cranial morphology of *Fukomys* and *Bathyergus* are congruent with their respective chisel-tooth and scratch digging behaviours and, all other factors being equal, would enable the more efficient production of bite force at wider gapes in *Fukomys*. However, *in vivo* measurements of muscle forces and activation patterns are needed to fully understand the complex biomechanics of tooth digging.

## 1. Introduction

The African mole-rats, or blesmols, are a family of rodents (Bathyergidae) comprising 25–30 species, all of which spend a large proportion of their life underground [1]. Of the six extant genera, five are chisel-tooth diggers, that is, they dig tunnels with their enlarged rodent incisors. Just one genus (*Bathyergus*) is a scratch digger, tunnelling with only its forelimbs and claws [2]. Chisel-tooth digging is a specialized form of tunnel construction that has also evolved independently in several other families of subterranean and fossorial rodents [3]. It is thought to have evolved in order to exploit harder soils as incisors are covered in hard enamel and fixed within the cranium and mandible. This is in contrast to the claws, which are made up of softer keratin and have more flexibility [4].

A number of morphological characteristics in the cranium have been associated with chisel-tooth digging. These include: more procumbent incisors, wider crania, enlarged zygomatic arches and larger temporal fossae [5–9]. Chisel-tooth digging mandibles are also convergent across rodents and show higher coronoid processes, reduced condyle heights and deep incisor roots [6,10,11]. Such traits have been linked to the requirement for chisel-tooth diggers to produce high bite force at the incisors at wide gape [5,6,10], and have also been found in carnivorans with similar functional requirements [12–14]. Within chisel-tooth digging species, variation in cranial morphology has been suggested to correlate with soil type, indicating that digging has a major influence on skull shape [15,16].

To understand how morphological traits can impact biomechanical function in extant and extinct vertebrates, many researchers have turned to the engineering technique finite-element analysis (FEA) over the decade or so [17–26]. FEA allows stress, strain and deformation to be predicted in a complex three-dimensional object subjected to a load, by dividing that object into a large number of smaller, simpler elements (usually cubes or tetrahedra) connected at nodes [27]. As a modelling technique, the results of FEA, and the conclusions that can be drawn from them, are necessarily limited by the accuracy of the model inputs. In particular, parameters such as material properties, constraints and loads are often unknown or can only be roughly estimated in biological models. Indeed, some validation studies have indicated that outputs from FEA (e.g. strain values) do not always match *ex vivo* or *in vivo* measurements in absolute terms [18,28,29]. However, these studies also indicate that the relative values are generally correct (e.g. areas of high strain and low strain predicted by FEA match those measured *in vivo*). Thus, while comparisons of absolute values between different unvalidated FE models can be difficult to interpret, comparisons between different loading scenarios in the same model (i.e. where one parameter is varied but all others are held constant) are justified.

The aim of this study is to predict the performance of the skull of two bathyergid mole-rats, one chisel-tooth digger and one scratch digger, when loaded at the incisors over a number of different gapes. It is hypothesized that the shape of the cranium of the chisel-tooth digging species will lead to improved performance at the incisors compared with the scratch digging species, particularly at wide gapes. FEA will be used to simulate masticatory muscle loading at different gape angles, and the patterns of stress distribution across the cranium will be predicted, as well as bite force at the incisors. By integrating geometric morphometrics (GMM) with FEA [22,30], it will also be possible to quantify and visualize the differences in overall deformations of the cranium between the two species. Following Dumont *et al*. [20], biomechanical performance will be measured in three ways. We predict that, compared with the scratch digging species, the chisel-tooth digging cranial model at wide gapes will: (i) exhibit lower stress (and thus be more resistant to structural failure); (ii) be more efficient at converting muscle forces to bite forces and (iii) experience less deformation. These predictions are based on the hypothesis that the cranial morphology of the chisel-tooth digging mole-rat will be adapted to both generate high forces at the incisors and withstand the reaction forces. It should be noted that this analysis seeks only to understand the impact of the difference in cranial morphology between *Fukomys* and *Bathyergus*. Many other factors can influence digging biomechanics, such as muscle physiology, muscle activation patterns and bone material properties, but data on these are scant in mole-rats and they are beyond the focus of this study.

## 2. Material and methods

### 2.1. Model construction

Finite-element (FE) models were created from microCT scans of two adult African mole-rat skulls: the chisel-tooth digging *Fukomys mechowii* (Muséum National d'Histoire Naturelle, Paris, ZM-MO-1911-664) and the scratch digging *Batherygus suillus* (Specimen 631, Professor Nigel Bennett, University of Pretoria). The specimens were scanned in an X-Tek Metris microCT scanner at the University of Hull (Medical and Biological Engineering Research Group). The scans had isometric voxels of 0.0417 mm (*Fukomys*) and 0.0532 mm (*Bathyergus*). Using Avizo v. 8.0 (FEI, Hillsboro, OR, USA), the scans were resampled to double their original voxel sizes to ensure a reasonable processing time during FE model creation and solving stages. Three-dimensional volume reconstructions of the skulls were created by a combination of automated and manual thresholding of materials. Bone, teeth and incisor pulp cavity were segmented as separate volumes, with all bone modelled as cortical bone. The reconstructions were then converted to an 8-noded cubic mesh directly from voxels using VOX-FE, in-house custom-built FEA software [31]. The *Fukomys* and *Bathyergus* models comprised 9 481 075 and 6 796 670 elements, respectively.

Based on previous nano-indentation work on rodents [21,32] and other mammals [19], bone and teeth were assigned Young's moduli of 17 and 30 GPa, respectively. Pulp was assigned a Young's modulus of 2 MPa [33]. All materials were modelled as homogeneous and isotropic with a Poisson's ratio of 0.3 being assigned to bone and teeth and a ratio of 0.45 to pulp [33]. No data are available for material properties of bathyergids. However, it was considered appropriate to use these properties as this study is primarily concerned with the relative digging performance between two species, and therefore is less concerned with absolute output values.

**Figure 1.**
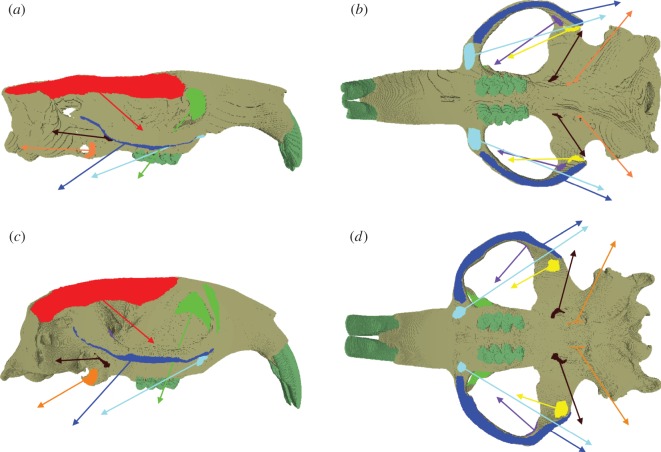
Attachment sites and vectors of pull of the masticatory muscles in models of *Bathyergus suillus*, in (*a*) right lateral and (*b*) ventral view and *Fukomys mechowii*, in (*c*) right lateral and (*d*) ventral view. Colours of muscle origins and vectors: temporalis, red; superficial masseter, cyan; deep masseter, royal blue; infraorbital ZM, green; anterior ZM, purple; posterior ZM, yellow; lateral pterygoid, brown; medial pterygoid, orange.

In order to model chisel-tooth digging, the models were constrained at the point of contact of the incisor tip with the substrate in the direction of the bite (i.e. orthogonal to the occlusal plane). Forty nodes were constrained at each temporo-mandibular joint (TMJ) in all three axis. Loads were added to the model representing the following muscles: temporalis, superficial masseter, deep masseter, zygomaticomandibularis (ZM: infraorbital, anterior and posterior parts), lateral pterygoid and medial pterygoid (figure 1). The masseter muscle was divided into three parts (superficial, deep and ZM) following [34,35]. Muscle attachment sites were assigned based on previously published dissections [36–38] and virtual muscle reconstructions [39] of bathyergids. Equal loads were applied to each side of the model as many rodents have demonstrated a bilateral muscle activation pattern when biting at the incisors [40,41].

The direction of pull of each muscle (i.e. muscle directional vector) was determined by placing a reconstruction of the specimen's mandible in a position of incisor occlusion (0°) with the cranial reconstruction using Avizo. Landmarks were placed at the centroid of each muscle attachment site on the mandible. These landmarks were then uploaded into VOX-FE to provide endpoints for the muscle direction vectors. The *Bathyergus* and *Fukomys* mandibles were automatically segmented in Avizo from microCT scans (0.0481 and 0.0350 mm isometric voxel sizes, respectively). To calculate muscle magnitudes, physiological cross-sectional area (PCSA) values for *F. mechowii* were taken from Van Daele *et al*. [38], and then multiplied by an intrinsic muscle stress value of 0.3 N mm^−2^ [42]. No PCSA data were available for *Bathyergus*, so the *Fukomys* muscle forces, scaled to model size, were used instead (the details and limitations of this are discussed below). Muscle loads for each model are given in table 1. To replicate different angles of gape, muscle directional vectors were rotated about an axis running between the left and right TMJ (see [6] for further details of method). Condyle translation has been shown to occur in the terrestrial rodent*, Pedetes capensis* during different stages of mastication [40]. However, condyle movement during digging at the incisors has been shown to be stable in *Ctenomys*, a South American subterranean rodent [11]. For this reason, condyle translation has not been included in the model, and the mandible has been simply rotated around an axis (TMJ).

**Table 1.** Muscle loads applied to each side of the FE models of *Fukomys mechowii* and *Bathyergus suillis*.

	force (N)	
muscle	*Fukomys*	*Bathyergus*
temporalis	20.7	24.1
superficial masseter	6.3	7.3
deep masseter	11.7	13.6
anterior ZM	1.5	1.7
posterior ZM	1.5	1.7
infraorbital ZM	3.0	3.5
medial pterygoid	5.1	6.0
lateral pterygoid	3.3	3.8

### 2.2. Analysis

In this study, von Mises (VM) stress was used as a key indicator of performance. Structures which exhibit overall lower VM stresses in a comparative context are less likely to fail under a given loading. If two models of the same shape but of different sizes have equal loads, the larger model will exhibit less stress (as stress equals force applied over the area of the model). To consider the effect of difference in shape on stress between two models, the effect of size must be controlled for, which can be achieved by keeping the ratio of force to surface area constant between the two models [43]. As PCSA values were not available for *Bathyergus*, surface areas for both models were calculated in Avizo, and the ratio of the two surface areas was used to scale forces applied to the *Bathyergus* model. Thus, the impact of cranial morphology on VM stress values for each model could be directly compared without the confounding influence of size. In order to quantify VM stress across the skull, the VM stress of each element from each model was extracted and the median VM stress for both models was calculated. Using the median, rather than the mean, to compare VM stress prevents outlying values that can arise from modelling artefacts from exaggerating the average stress value.

The mechanical efficiency of incisor biting in each model was also calculated to assess the performance of both models. Mechanical efficiency is the ratio of predicted bite force to total muscle input force and provides a single value, independent of size, to assess the efficiency of the masticatory system in transforming muscle to bite force [20,21]. Absolute bite force was not reported in this study as the muscle forces and geometry of the *Fukomys* model were gleaned from separate specimens. Therefore, there is no expectation that the bite force will be biologically accurate. However, dividing bite force by input muscle force to produce mechanical efficiency produces a meaningful performance parameter for comparison between FE models.

**Figure 2.**
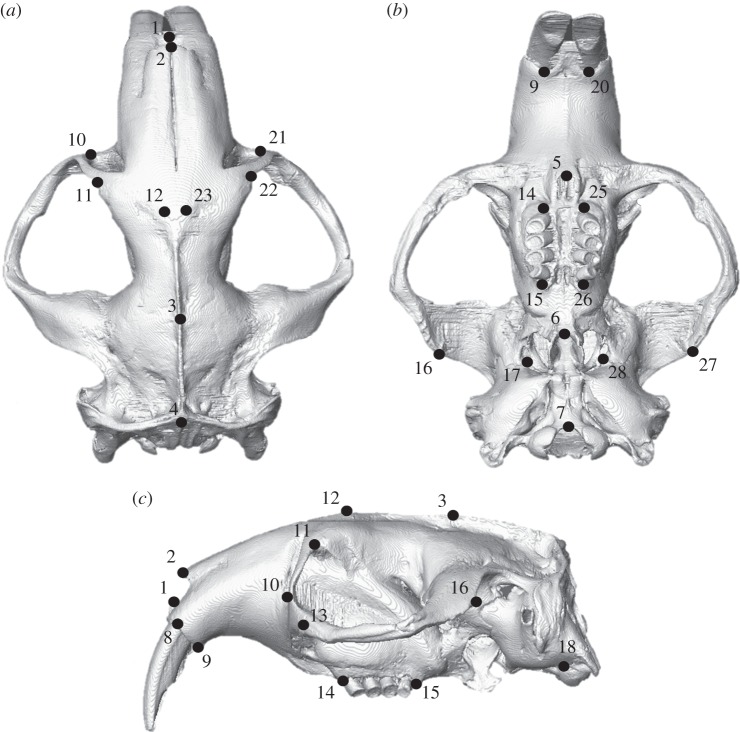
Landmark configuration represented on *Fukomys mechowii* in (*a*) dorsal, (*b*) ventral and (*c*) left lateral view. Text definitions of landmarks given in the electronic supplementary material, table S1.

GMM was used to analyse variation in deformations between the FE models, following [22,23,26,30,31,44]. A set of three-dimensional landmarks (figure 2 and electronic supplementary material, table S1) were recorded from the unloaded and loaded models. The landmarks were then subjected to a generalized Procrustes analysis and scaled to centroid size. The residual differences between the loaded and unloaded models were then added to the mean landmark configuration of the unloaded *Fukomys* and *Bathyergus* models. The mean and loaded configurations were subjected to a second Procrustes analysis without scaling or tangent projection [44], to represent the multivariate data on a graph. Cranial deformations were visualized via surface rendering of a hybrid of the two unloaded models warped along the vectors of deformation. Deformations were magnified 500 times to aid visual interpretation of transformation grids. All GMM analyses were carried out using the EVAN toolbox (www.evan-society.org). Further details of the GMM methods and the theory underlying them are given in the electronic supplementary material, text S1.

It should be noted that because no muscle PCSA data was available for the individuals from which the model geometries were constructed, the resulting data should not be treated as reflecting biological reality. The scaling of the muscle forces instead allows us to draw conclusions on the relative impact of changing muscle orientations in species with different cranial morphologies.

## 3. Results

**Figure 3.**
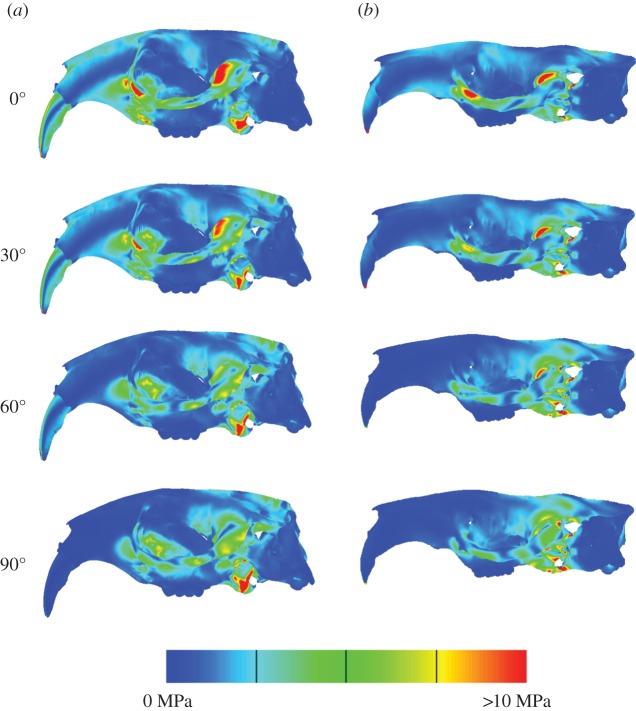
Predicted von Mises stress distributions across the skulls of *Fukomys* (*a*) and *Bathyergus* (*b*) during incisor biting at four different gape angles.

Figure 3 shows the distribution of VM stress across the crania of the two models. As might be expected, at occlusion both models show areas of high stress around the constraints (glenoid fossae and incisor tips), and some of the muscle attachment sites (zygomatic arch and pterygoid fossa). Beyond these areas, both models also show high stresses in the postero-ventral part of the rostrum. In addition, the *Fukomys* cranium has high stresses in the dorsal rostrum and in the incisor itself. As gape increases, stress tends to decrease in the rostrum and anterior zygomatic arch, and increase in the temporal region and posterior orbital region. Studying median VM stresses (table 2) shows that increasing gape reduces the overall stress in the cranium, and that *Fukomys* experiences higher VM stress in the cranium at each pairwise gape compared with *Bathyergus*.

**Table 2.** Median von Mises stress and mechanical efficiency of biting in *Fukomys mechowii* and *Bathyergus suillis* at increasing gape.

	median VM (MPa)		mechanical efficiency	
gape angle (°)	*Fukomys*	*Bathyergus*	*Fukomys*	*Bathyergus*
0	1.06	0.88	0.18	0.13
30	1.04	0.76	0.15	0.08
60	0.85	0.52	0.09	0.03
90	0.63	0.59	0.02	−0.03

The mechanical efficiency of biting (the ratio of predicted bite force to input muscle force) at each gape in the two species is given in table 2. *Fukomys* is more efficient than *Bathyergus* at converting input forces to output forces at all gape angles. As gape increases, mechanical efficiency decreases in both specimens, but at different rates. Specifically, *Fukomys* is half as efficient at 60° as at 0°, whereas the mechanical efficiency of *Bathyergus* at 60° is only a quarter of its efficiency at occlusion. It should be noted that the mechanical efficiency (and thus bite force) of *Bathyergus* at 90° gape is negative. This is a result of many of the muscle vectors rotating so far around that they now exert an upward rather than downward force on the skull model and is clearly a biologically unrealistic situation.

**Figure 4.**
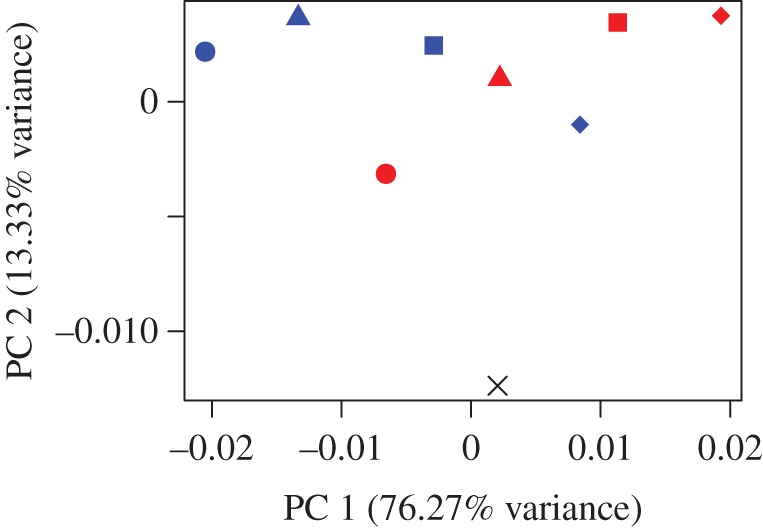
Principal component analysis (PCA) plot representing the differences of deformations between the two models scaled to force : area ratio. Cross, mean unloaded model; blue shapes, *Fukomys* models; red shapes, *Bathyergus* models; circles, occlusion; triangles, 30° gape; squares, 60° gape; diamonds, 90° gape.

**Figure 5.**
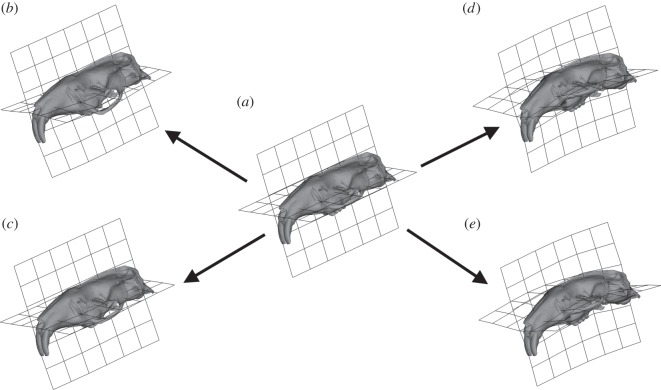
Transformation grids and surface warps associated with PCA plot (figure 4) representing the differences of deformation between the two models scaled to force : area ratio. Arrows represent the change in size and shape between unloaded mean model and target. (*a*) Unloaded mean model, (*b*) size and shape change from unloaded model to *Fukomys* model in occlusion, (*c*) size and shape change from unloaded model to *Bathyergus* model in occlusion, (*d*) size and shape change from unloaded model to *Fukomys* model at 90° gape and (*e*) size and shape change from unloaded model to *Bathyergus* model at 90° gape.

Figure 4 shows the size and shape deformations between the two model types at varying degrees of gape. PC1 represents 76.27% variance and PC2 13.33%. PC1 is dominated by the differences between the loaded models at differing angles of gape while PC2 shows the difference between the unloaded mean and the loaded models. *Bathyergus* in occlusion and *Fukomys* at 90° gape are the least deformed from the mean unloaded model; whereas *Bathyergus* at 90° gape and *Fukomys* in occlusion are the most deformed from the mean unloaded model. Figure 5*a*–*c* shows the deformation between the mean unloaded model and the two models at occlusion using thin plate splines. The main difference between the mean unloaded model and the loaded models at occlusion is the ventral deflection of the zygomatic arch. Figure 5*d*,*e* shows cranial deformations from unloaded mean to 90° gape in both models. The deformations between the two models are shown to be rather similar, with increasing gapes being associated with dorsoventral bending.

## 4. Discussion

### 4.1. Von Mises stress

The results of the FEA allow us to compare the biomechanical performance of the skull between gapes within each model. Three performance metrics were studied: median VM stress across the model, the ratio of predicted bite force to total input adductor muscle force and overall deformation of the model (following [20]). In both models, average VM stress decreases as gape increases (table 2). In particular, VM stress is reduced in the anterior part of the skull (figure 3), probably as a result of the muscle vectors being oriented in a more posterior, rather than ventral, direction. At each gape, the *Bathyergus* model experiences a lower median stress than the *Fukomys* model, suggesting that the morphology of the *Bathyergus* cranium is better able to resist the forces applied to it in this analysis. This is counter to the first hypothesis that suggested the chisel-tooth digging species would exhibit lower stresses at wider gapes. However, we urge caution in interpreting this result as, although the muscle forces were scaled to surface area to enable direct comparisons of stress values [43], it only indicates how the cranial morphology responds to forces. In reality, there are likely to be large differences in the muscle force to surface area ratio, as well as potential differences in the relative proportions of the muscles and the bone material properties between the two taxa.

It is unclear whether VM stress values really matter in an evolutionary context, as long as they are below the yield strength of bone, and there is little evidence of cranial bone naturally loading to failure [20]. Thus, assuming cranial stress is within a suitable safety factor, its precise value may not be important. Previous work has suggested that bats adapt their crania in favour of mechanical efficiency of biting, whereas adaptation to cranial strength (i.e. low VM stress) is not as strongly selected for [45].

### 4.2. Mechanical efficiency of biting

From table 2, it can be seen that the *Fukomys* model has a greater mechanical efficiency of biting than the *Bathyergus* model at all simulated gapes, not just at wider gapes as predicted in our second hypothesis. As would be expected from simple mechanics, bite force (and thus mechanical efficiency) decreased with increasing gape in both models [46–49]. However, the relative decrease with increasing gape was much greater in the *Bathyergus* model. That is, the cranial morphology of *Fukomys* is better able to maintain mechanical efficiency as the muscle forces rotate posteriorly. A higher mechanical efficiency can be partly achieved by having masticatory muscles that have increased moment arms around the TMJ. Previous work has indicated that the temporalis muscles of chisel-tooth digging bathyergids have increased moment arms compared with *Bathyergus* [6], and therefore this could be the muscle driving improved mechanical efficiency at increased gapes in the *Fukomys* model presented here.

### 4.3. Cranial deformation

The GMM analysis shows that the relative deformation of the models at different gapes follows an almost symmetrical pattern (figure 4). The main difference between the two models is that, as gape increases, the *Fukomys* model deforms less (plots closer to the unloaded model) and the *Bathyergus* model deforms more (plots further from the unloaded model). This result is as predicted by the third hypothesis and fits with the digging behaviour of these two species. It appears that in *Fukomys* the morphology of the cranium leads to reduced deformation at the wide gapes necessary for chisel-tooth digging [6,10]. *Bathyergus*, as a scratch digger [2], does not employ such wide gapes as frequently, and thus its cranial morphology deforms least at narrower gapes.

When comparing the two models at occlusion, it can be seen that the main difference in deformation occurs at the zygomatic arch, which is more ventrally deflected in *Fukomys* than *Bathyergus* (figure 5*b*,*c*). As the models have been scaled to the same muscle force : surface area ratio, it is unlikely that the greater zygomatic deformation is a product of greater muscle force in *Fukomys*; rather, it is differences in the direction of muscle pull that appear to be leading to this result. It can be seen in figure 1 that the deep masseter of *Bathyergus* has a greater posterior component to its line of action than does that of *Fukomys*. Thus, the forces acting on the zygomatic arch of *Fukomys* are likely to produce a greater ventral deflection than is seen in *Bathyergus*.

Figure 5*d*,*e* represents how the models deform at large gape angles. Both models seem to experience dorsoventral bending of the cranium. As gape increases, the arrangement of the most dominant muscles (figure 1), the temporalis (which attaches to the posterior area of the cranium) and masseters (which attach to the zygoma), will cause dorsoventral bending of the cranium around the TMJ constraints. Less bending will occur at the incisor as the muscle vectors rotate with the mandible as gape increases. This results in the muscle vectors directing more force towards the posterior part of the skull, and less force towards the anterior portion (this is also demonstrated by VM stress patterns in figure 3 where cranial stress is concentrated at the posterior areas of the cranium as gape increases). Interestingly, the *Fukomys* model does not experience as much deformation or dorsoventral bending at 90° gape compared with *Bathyergus* (figures 4 and 5*d*,*e*). This implies that the *Fukomys* cranium is stiffer than the *Bathyergus* cranium, which is to be expected from a cranium that has higher mechanical efficiency (table 2). The stiffer the cranium is during mastication, the less energy it will waste in deforming, making it more efficient at converting muscle forces into bite forces.

### 4.4. Conclusion

The results here demonstrate that the cranial morphology of *Fukomys* performs better during incisor biting at wide gapes than does *Bathyergus*. That is, the *Fukomys* model had a greater mechanical efficiency of biting than *Bathyergus* and was able to maintain it to a greater degree as gape increased. In addition, deformations of the *Fukomys* cranial model were smaller at larger gapes, whereas in *Bathyergus* deformations were smaller at narrower gapes. The relative performance of the models is congruent with the known digging behaviour of the two species under study here, i.e. chisel-tooth digging in *Fukomys* and scratch digging in *Bathyergus* [2]. Previous studies of subterranean rodents have indicated that digging behaviour has a major impact on cranial morphology [15,16] and that chisel-tooth digging species have adaptations for high bite force and wide gape [6,10]. The cranial morphology of the chisel-tooth digger in this analysis is clearly able to function well at wide gapes, and, although absolute bite force cannot be predicted with any degree of confidence by our unvalidated models, increasing the efficiency of the masticatory system would necessarily increase bite force. It should be emphasized that the conclusions drawn here relate only to the morphology of the cranium. To understand the biomechanics of digging more thoroughly would require a much more complex model incorporating data on muscle physiology, bone material properties, behaviour and many other factors, which we feel would be a very fruitful avenue of research.

## Supplementary Material

Table S1: Cranial landmarks used in geometric morphometric analysis.

## Supplementary Material

Text S1: Details of geometric morphometric methods.

## Data accessibility

Surface reconstructions, FE models and displacement files available from https://figshare.com/articles/Mole_rat_FEA/3188830.

## References

[1] Faulkes CG, Bennett NC 2013 Plasticity and constraints on social evolution in African mole-rats: ultimate and proximate factors. Phil. Trans. R. Soc. B 368, 20120347 (doi:10.1098/rstb.2012.0347) 23569295 10.1098/rstb.2012.0347 PMC3638450

[2] Jarvis JUM, Bennett NC 1991 Ecology and behaviour of the family Bathyergidae. In The biology of the naked mole-rat (eds Sherman PW, Jarvis JUM, Alexander RD), pp. 66–96. Princeton, NJ: Princeton University Press.

[3] Stein BR 2000 Morphology of subterranean rodents. In Life underground: the biology of subterranean rodents (eds Lacey EA, Patton JL, Cameron GN), pp. 19–61. Chicago, IL: University of Chicago Press.

[4] Lessa EP, Thaeler CS 1989 A reassessment of morphological specializations for digging in pocket gophers. J. Mammal. 70, 689–700. (doi:10.2307/1381704)

[5] Samuels JX, Van Valkenburgh B 2009 Craniodental adaptations for digging in extinct burrowing beavers. J. Vert. Paleo. 29, 254–268. (doi:10.1080/02724634.2009.10010376)

[6] McIntosh AF, Cox PG 2016 Functional implications of craniomandibular morphology in African mole-rats (Rodentia: Bathyergidae). Biol. J. Linn. Soc. 117, 447–462. (doi:10.1111/bij.12691)

[7] Landry SO 1957 Factors affecting the procumbency of rodent upper incisors. J. Mammal. 38, 223–234. (doi:10.2307/1376314)

[8] Agrawal V 1967 Skull adaptations in fossorial rodents. Mammalia 31, 300–312. (doi:10.1515/mamm.1967.31.2.300)

[9] Lessa EP, Stein BR 1992 Morphological constraints in the digging apparatus of pocket gophers (Mammalia: Geomyidae). Biol. J. Linn. Soc. 47, 439–453. (doi:10.1111/j.1095-8312.1992.tb00678.x)

[10] Gomes Rodrigues H, Šumbera R, Hautier L 2016 Life in burrows channelled the morphological evolution of the skull in rodents: the case of African mole-rats (Bathyergidae, Rodentia). J. Mamm. Evol. 23, 175–189. (doi:10.1007/s10914-015-9305-x)

[11] Verzi DH, Olivares AI 2006 Craniomandibular joint in South American burrowing rodents (Ctenomyidae): adaptations and constraints related to a specialized mandibular position in digging. J. Zool. 270, 488–501. (doi:10.1111/j.1469-7998.2006.00167.x)

[12] Wroe S, McHenry C, Thomason J 2005 Bite club: comparative bite force in big biting mammals and the prediction of predatory behaviour in fossil taxa. Proc. R. Soc. B 272, 619–625. (doi:10.1098/rspb.2004.2986) 10.1098/rspb.2004.2986 PMC1564077 15817436

[13] Wroe S, Milne N 2007 Convergence and remarkably consistent constraint in the evolution of carnivore skull shape. Evolution 61, 1251–1260. (doi:10.1111/j.1558-5646.2007.00101.x) 17492976 10.1111/j.1558-5646.2007.00101.x

[14] Figueirido B, Tseng ZJ, Martín-Serra A 2013 Skull shape evolution in durophagous carnivorans. Evolution 67, 1975–1993. (doi:10.1111/evo.12059) 23815654 10.1111/evo.12059

[15] Barčiová L, Šumbera R, Burda H 2009 Variation in the digging apparatus of the subterranean silvery mole-rat *Heliophobius argenteocinereus* (Rodentia, Bathyergidae): the role of ecology and geography. Biol. J. Linn. Soc. 97, 822–831. (doi:10.1111/j.1095-8312.2009.01228.x)

[16] Beolchini F, Corti M 2004 The taxonomy of the genus *Tachyoryctes*: a geometric morphometric approach. Ital. J. Zool. 71, 35–43. (doi:10.1080/11250000409356548)

[17] Rayfield EJ 2004 Cranial mechanics and feeding in *Tyrannosaurus rex*. Proc. R. Soc. Lond. B 271, 1451–1459. (doi:10.1098/rspb.2004.2755) 10.1098/rspb.2004.2755 PMC1691752 15306316

[18] Strait DS, Wang Q, Dechow PC, Ross CF, Richmond BG, Spencer MA, Patel BA 2005 Modeling elastic properties in finite-element analysis: how much precision is needed to produce an accurate model? Anat. Rec. 283A, 275–287. (doi:10.1002/ar.a.20172) 10.1002/ar.a.20172 15747346

[19] Kupczik K, Dobson CA, Fagan MJ, Crompton RH, Oxnard CE, O'Higgins P 2007 Assessing mechanical function of the zygomatic region in macaques: validation and sensitivity testing of finite element models. J. Anat. 210, 41–53. (doi:10.1111/j.1469-7580.2006.00662.x) 17229282 10.1111/j.1469-7580.2006.00662.x PMC2100262

[20] Dumont ER, Davis JL, Grosse IR, Burrows AM 2011 Finite element analysis of performance in the skulls of marmosets and tamarins. J. Anat. 218, 151–162. (doi:10.1111/j.1469-7580.2010.01247.x) 20572898 10.1111/j.1469-7580.2010.01247.x PMC3039787

[21] Cox PG, Rayfield EJ, Fagan MJ, Herrel A, Pataky TC, Jeffery N 2012 Functional evolution of the feeding system in rodents. PLoS ONE 7, e36299 (doi:10.1371/journal.pone.0036299) 22558427 10.1371/journal.pone.0036299 PMC3338682

[22] O'Higgins P, Fitton L, Phillips R, Shi J, Liu J, Gröning F, Cobb S, Fagan MJ 2012 Virtual functional morphology: novel approaches to the study of craniofacial form and function. Evol. Biol. 39, 521–535. (doi:10.1007/s11692-012-9173-8)

[23] Cox PG, Kirkham J, Herrel A 2013 Masticatory biomechanics of the Laotian rock rat, *Laonastes aenigmamus*, and the function of the zygomaticomandibularis muscle. PeerJ 1, e160 (doi:10.7717/peerj.160) 24058888 10.7717/peerj.160 PMC3775629

[24] O'Hare LMS, Cox PG, Jeffery N, Singer ER 2013 Finite element analysis of stress in the equine proximal phalanx. Equine Vet. J. 45, 273–277. (doi:10.1111/j.2042-3306.2012.00635.x) 22943561 10.1111/j.2042-3306.2012.00635.x

[25] Cox PG, Rinderknecht A, Blanco RE 2015 Predicting bite force and cranial biomechanics in the largest fossil rodent using finite element analysis. J. Anat. 226, 215–223. (doi:10.1111/joa.12282) 25652795 10.1111/joa.12282 PMC4337660

[26] Fitton LC, Prôa M, Rowland C, Toro-Ibacache V, O'Higgins P 2015 The impact of simplifications on the performance of a finite element model of a *Macaca fascicularis* cranium. Anat. Rec. 298, 107–121. (doi:10.1002/ar.23075) 10.1002/ar.23075 25339306

[27] Rayfield EJ 2007 Finite element analysis and understanding the biomechanics and evolution of living and fossil organisms. Annu. Rev. Earth Planet. Sci. 35, 541–576. (doi:10.1146/annurev.earth.35.031306.140104)

[28] Bright JA, Rayfield EJ 2011 Sensitivity and *ex vivo* validation of finite element models of the domestic pig cranium. J. Anat. 219, 456–471. (doi:10.1111/j.1469-7580.2011.01408.x) 21718316 10.1111/j.1469-7580.2011.01408.x PMC3196751

[29] Porro LB, Metzger KA, Iriarte-Diaz J, Ross CF 2013 *In vivo* bone strain and finite element modelling of the mandible of *Alligator mississippiensis*. J. Anat. 223, 195–227. (doi:10.1111/joa.12080) 23855772 10.1111/joa.12080 PMC3972043

[30] O'Higgins P, Cobb SN, Fitton LC, Gröning F, Phillips R, Liu J, Fagan MJ 2011 Combining geometric morphometrics and functional simulation: an emerging toolkit for virtual functional analyses. J. Anat. 218, 3–15. (doi:10.1111/j.1469-7580.2010.01301.x) 20880075 10.1111/j.1469-7580.2010.01301.x PMC3039776

[31] Liu J, Shi J, Fitton L, Phillips R, O'Higgins P, Fagan MJ 2012 The application of muscle wrapping to voxel-based finite element models of skeletal structures. Biomech. Model. Mechanobiol. 11, 35–47. (doi:10.1007/s10237-011-0291-5) 21308392 10.1007/s10237-011-0291-5

[32] Cox PG, Fagan MJ, Rayfield EJ, Jeffery N 2011 Finite element modelling of squirrel, guinea pig and rat skulls: using geometric morphometrics to assess sensitivity. J. Anat. 219, 696–709. (doi:10.1111/j.1469-7580.2011.01436.x) 21974720 10.1111/j.1469-7580.2011.01436.x PMC3237878

[33] Williams KR, Edmundson JT 1984 Orthodontic tooth movement analysed by the finite element method. Biomaterials 5, 347–351. (doi:10.1016/0142-9612(84)90033-4) 6525394 10.1016/0142-9612(84)90033-4

[34] Turnbull WD 1970 Mammalian masticatory apparatus. *Fieldiana* (Geol.) 18, 147–356.

[35] Cox PG, Jeffery N 2011 Reviewing the morphology of the jaw-closing musculature in squirrels, rats, and guinea pigs with contrast-enhanced microCT. Anat. Rec. 294, 915–928. (doi:10.1002/ar.21381) 10.1002/ar.21381 21538924

[36] Boller N 1970 Untersuchungen an Schädel, Kaumuskulatur und äußerer Hirnform von *Cryptomys hottentotus* (Rodentia, Bathyergidae). Z. wiss. Zool. 181, 7–65.

[37] Morlok WF 1983 Vergleichend- und funktionell-anatomische Untersuchungen an Kopf, Hals und Vorderextremität subterraner Nagetiere (Mammalia, Rodentia). Cour. Forsch. Inst. Senck. 64, 1–237.

[38] Van Daele P, Herrel A, Adriaens D 2009 Biting performance in teeth-digging African mole-rats (*Fukomys*, Bathyergidae, Rodentia). Physiol. Biochem. Zool. 82, 40–50. (doi:10.1086/594379) 19035771 10.1086/594379

[39] Cox PG, Faulkes CG 2014 Digital dissection of the masticatory muscles of the naked mole-rat, *Heterocephalus glaber* (Mammalia, Rodentia). PeerJ 2, e448 (doi:10.7717/peerj.448) 25024917 10.7717/peerj.448 PMC4081180

[40] Offermans M, de Vree F 1990 Mastication in springhares, *Pedetes capensis*: a cineradiographic study. J. Morphol. 205, 353–367. (doi:10.1002/jmor.1052050310) 10.1002/jmor.1052050310 29865740

[41] Satoh K 1998 Balancing function of the masticatory muscles during incisal biting in two murid rodents, *Apodemus speciosus* and *Clethrionomys rufocanus*. J. Morphol. 236, 49–56. (doi:10.1002/(SICI)1097-4687(199804)236:1<49::AID-JMOR3>3.0.CO;2-J) 9503662 10.1002/(SICI)1097-4687(199804)236:1<49::AID-JMOR3>3.0.CO;2-J

[42] Weijs W, Hillen B 1985 Cross-sectional areas and estimated intrinsic strength of the human jaw muscles. Acta Morphol. Neerl. Scand. 23, 267–274. 4096273

[43] Dumont ER, Grosse IR, Slater GJ 2009 Requirements for comparing the performance of finite element models of biological structures. J. Theor. Biol. 256, 96–103. (doi:10.1016/j.jtbi.2008.08.017) 18834892 10.1016/j.jtbi.2008.08.017

[44] O'Higgins P, Milne N 2013 Applying geometric morphometrics to compare changes in size and shape arising from finite elements analyses. Hystrix 24, 126–132.

[45] Dumont ER, Samadevam K, Grosse I, Warsi OM, Baird B, Davalos LM 2014 Selection for mechanical advantage underlies multiple cranial optima in new world leaf-nosed bats. Evolution 68, 1436–1449. (doi:10.1111/evo.12358) 24433457 10.1111/evo.12358

[46] Herring SW, Herring SE 1974 The superficial masseter and gape in mammals. Am. Nat. 108, 561–576. (doi:10.1086/282934)

[47] Dumont ER, Herrel A 2003 The effects of gape angle and bite point on bite force in bats. J. Exp. Biol. 206, 2117–2123. (doi:10.1242/jeb.00375) 12771161 10.1242/jeb.00375

[48] Bourke J, Wroe S, Moreno K, McHenry C, Clausen P 2008 Effects of gape and tooth position on bite force and skull stress in the dingo (*Canis lupus dingo*) using a 3-dimensional finite element approach. PLoS ONE 3, e2200 (doi:10.1371/journal.pone.0002200) 18493603 10.1371/journal.pone.0002200 PMC2376057

[49] Williams SH, Peiffer E, Ford S 2009 Gape and bite force in the rodents *Onychomys leucogaster* and *Peromyscus maniculatus*: does jaw-muscle anatomy predict performance? J. Morphol. 270, 1338–1347. (doi:10.1002/jmor.10761) 19480012 10.1002/jmor.10761

