# Supplementary material for: The impact of gape on the performance of the skull in chisel-tooth digging and scratch digging mole-rats (Rodentia: Bathyergidae)
Source: R Soc Open Sci. 2016 Oct 12;3(10):160568. doi: 10.1098/rsos.160568 (PMC5099000; doi:10.1098/rsos.160568)
Supplement: Table S1: Cranial landmarks used in geometric morphometric analysis. [file rsos160568supp1.docx]

**Table S1.** Cranial landmarks used in GMM analysis.

| **Number** | **Landmark definition** |
| --- | --- |
| 1 | Midpoint of ventral margin of nasal opening |
| 2 | Anteriormost point on internasal suture |
| 3 | Bregma |
| 4 | Posteriormost point on dorsal midline |
| 5 | Midpoint between anterior extremities of incisive foramina |
| 6 | Posteriormost midline point on palatine |
| 7 | Midline point on ventral margin of foramen magnum |
| 8 & 19 | Dorsalmost point on incisal alveolar margin |
| 9 & 20 | Posteriormost point on incisal alveolar margin |
| 10 & 21 | Lateralmost point on margin of infraorbital foramen |
| 11 & 22 | Dorsalmost point on orbital margin |
| 12 & 23 | Posteriormost point of naso-frontal suture |
| 13 & 24 | Anteriormost point of maxillo-jugal suture |
| 14 & 25 | Anterior extremity of cheek tooth row |
| 15 & 26 | Posterior extremity of cheek tooth row |
| 16 & 27 | Posterior tip of zygomatic arch |
| 17 & 28 | Posteriormost point of foramen ovale |
| 18 & 29 | Lateralmost point of hypoglossal foramen |

Landmarks 1-7 recorded from midline, landmarks 8-18 recorded on left side of skull, landmarks 19-29 recorded on right side of skull.
