# Supplementary material for: The impact of gape on the performance of the skull in chisel-tooth digging and scratch digging mole-rats (Rodentia: Bathyergidae)
Source: R Soc Open Sci. 2016 Oct 12;3(10):160568. doi: 10.1098/rsos.160568 (PMC5099000; doi:10.1098/rsos.160568)
Supplement: Text S1: Details of geometric morphometric methods. [file rsos160568supp2.docx]

**Text S1**

*Details of geometric morphometric methods*

When ductile materials have forces applied to them, they will elastically deform. The further a loaded object is from its original form, the more likely it is to plastically deform, and so an object’s global deformation is of particular interest to biologists trying to assess the performance of morphology during different loading conditions. Visually, it can be rather difficult to assess variation between FE models as deformations between unloaded and loaded models are small. Recently, geometric morphometrics (GMM), a landmark based form analysis method (for review, see O'Higgins, 2000) has been applied to finite element analyses in order to quantify global deformations between loaded and unloaded models (O’Higgins et al., 2011, 2012; Cox et al., 2011; Gröning et al., 2011; O'Higgins and Milne, 2013; Fitton et al., 2015). It should also be noted that GMM analyses cannot predict material failures (which is done by strain analysis), as they can only quantify the differences of size and shape between the deformations, and not the processes that achieve these deformations (O'Higgins and Milne, 2013). Therefore, the term deformation used throughout refers to differences in size and shape of models and not rigid body motion (as rigid body displacement is removed during GMM analysis). A set of landmarks (3-D coordinate data) were placed onto selected nodes of each unloaded model (Figure 1). The landmarks were then extracted from displaced nodes from the loaded models and analysed to quantify the size and shape difference between the unloaded and loaded models.

The majority of GMM analyses concentrate on the shape of an object and as such ‘remove’ the size aspect of an object. This is achieved by scaling all configurations to unit centroid size: the square root of the sum of squared landmark differences to the centroid. Each landmark configuration is also translated to an arbitrary axis coordinate system. Each configuration is then rotated with respect to an average configuration in order to minimize the distance between corresponding landmarks. This process is called a generalized Procrustes analysis (Dryden and Mardia, 1998) and the resulting landmark coordinates (Procrustes coordinates) are now represented on Kendall’s shape space (Kendall, 1984). However, deformations that occur in FEA have arisen due to differences in size as well as shape. In a mechanical context, it makes little sense to attribute deformations to shape alone (O’Higgins and Milne, 2013). Past studies that have applied GMM to FEA circumvent this problem by not scaling each landmark configuration to its centroid size during the Procrustes procedure, minimizing the landmark differences between unscaled configurations (Milne and O'Higgins, 2012; O'Higgins and Milne, 2013, Fitton et al., 2015). This method represents the Procrustes coordinates in “size and shape” space (Dryden and Mardia, 1998; Dryden et al., 2007) instead of Kendall’s shape space. This is a potential problem, as methods that are not based on Kendall’s shape space have been shown to strongly constrain the possible results obtained by ordination methods (Rohlf, 2000). To overcome this issue, landmark coordinates (of both unloaded models and all loaded models) were subjected to a full Procrustes analysis and were not projected onto a tangent space in order to keep the coordinates (Procrustes) on the surface of Kendall’s shape space (see Rohlf, 1999 for more information on this). To include the effect of size, the Procrustes coordinates of each model were then scaled to their respective centroid sizes.

In order to quantify the differences in deformation between two specimens in a comparative analysis, the difference in size and shape between the two unloaded models must be discarded, as these differences will far outweigh the differences found between the unloaded and loaded models (Fitton et al., 2015). To compare how two different models deform, both models’ landmark coordinates (unloaded and loaded) are subjected to the same Procrustes analysis and scaled according to their centroid sizes (as above). The difference in Procrustes coordinates (after centroid size scaling) between each loaded and unloaded model were calculated to obtain their residuals (these residuals represent the displacement of selected nodes on each model after the FE analysis). In order to visualise the differences in deformations between the two models, the residuals were added to a mean unloaded landmark configuration, which is calculated from the two unloaded model configurations after Procrustes fitting and scaling (from the above analysis). This mean configuration (which represents the mean unloaded model) and the loaded models’ configurations (mean unloaded model plus each loaded models’ residuals) are subjected to a second Procrustes analysis, without scaling or tangent projection (O’Higgins and Milne, 2013), essentially carrying out a principal components analysis on the above coordinates to represent the multivariate data on a graph. Size and shape differences between the mean model and the loaded models were visualized firstly by warping the *Bathyergus* surface (using Avizo) to the mean configuration of the unloaded models, therefore creating a *Bathyergus-Fukomys* hybrid surface. This hybrid surface was then used together with transformation grids, calculated via the thin plate spline (TPS) method (Bookstein, 1989) to visualize the size and shape deformations. As these deformations are very small, to aid visual interpretation, the deformations are magnified 500 times. This therefore means the resulting bending energy represented on the transformation grid is not absolute, but is rather used as a visual device (O'Higgins and Milne, 2013).

*References*

Bookstein FL. 1989. Principal warps: thin-plate splines and the decomposition of deformations. *IEEE T Pattern Anal* 11**,** 567-585.

Cox PG, Fagan MJ, Rayfield EJ, Jeffery N. 2011. Finite element modelling of squirrel, guinea pig and rat skulls: using geometric morphometrics to assess sensitivity. *J Anat* 219**,** 696-709.

Dryden I, Mardia K. 1998. *Statistical analysis of shape*. New York, NY: Wiley.

Dryden IL, Hirst JD, Melville JL. 2007. Statistical analysis of unlabeled point sets: comparing molecules in chemoinformatics. *Biometrics* 63**,** 237-251.

Fitton LC, Prôa M, Rowland C, Toro-Ibacache V, O'Higgins P. 2015. The impact of simplifications on the performance of a finite element model of a *Macaca fascicularis* cranium. *Anat Rec* 298**,** 107-121.

Gröning F, Fagan MJ, O’Higgins P. 2011. The effects of the periodontal ligament on mandibular stiffness: a study combining finite element analysis and geometric morphometrics. *J Biomech* 44**,** 1304-1312.

Kendall DG. 1984. Shape manifolds, procrustean metrics, and complex projective spaces. *Bull Lond Math Soc* 16**,** 81-121.

Milne N, O'Higgins P. 2012. Scaling of form and function in the xenarthran femur: a 100-fold increase in body mass is mitigated by repositioning of the third trochanter. *Proc R Soc B* 279**,** 3449-3456.

O'Higgins P. 2000. The study of morphological variation in the hominid fossil record: biology, landmarks and geometry. *J Anat* 197**,** 103-120.

O’Higgins P, Cobb SN, Fitton LC, Gröning F, Phillips R, Liu J, Fagan MJ. 2011. Combining geometric morphometrics and functional simulation: an emerging toolkit for virtual functional analyses. *J Anat* 218**,** 3-15.

O’Higgins P, Fitton L, Phillips R, Shi J, Liu J, Gröning F, Cobb S, Fagan MJ. 2012. Virtual functional morphology: novel approaches to the study of craniofacial form and function. *Evol Biol* 39**,** 521-535.

O'Higgins P, Milne N. 2013. Applying geometric morphometrics to compare changes in size and shape arising from finite elements analyses. *Hystrix* 24**,** 126-132.

Rohlf FJ. 1999. Shape statistics: procrustes superimpositions and tangent spaces. *J Classif* 16**,** 197-223.

Rohlf FJ. 2000. On the use of shape spaces to compare morphometric methods. *Hystrix* 11, 9-25.
